# Association between postoperative urinary incontinence severity and anxiety in patients with prostate cancer: A chain mediation analysis of ehealth literacy and pelvic floor muscle training adherence

**DOI:** 10.1016/j.apjon.2025.100842

**Published:** 2025-12-23

**Authors:** Qiuxia Qin, Juan Liu, Duo Zhang, Yuming Zhang, Xiaoqin Xie, Fan Yang, Lihong Huang, Xiaoqin Chen

**Affiliations:** Department of Nursing, Tongji Hospital, Tongji Medical College, Huazhong University of Science and Technology, Wuhan, China

**Keywords:** Prostate cancer, Postoperative urinary incontinence, Anxiety, eHealth literacy, Pelvic floor muscle training, Mediation analysis

## Abstract

**Objective:**

This study aimed to examine the chain mediating roles of eHealth literacy and pelvic floor muscle training (PFMT) adherence in the association between urinary incontinence (UI) severity and anxiety.

**Methods:**

A cross-sectional study was conducted using convenience sampling to recruit 240 patients with prostate cancer one month after surgery. Validated instruments were used to assess PFMT adherence (PFMT Adherence Scale), eHealth literacy (eHEALS), UI severity (International Consultation on Incontinence Questionnaire-Short Form), and anxiety (Generalized Anxiety Disorder-7). Structural equation modeling (SEM) was performed using AMOS 24.0 to evaluate direct and indirect effects. Mediation effects were tested using bias-corrected bootstrap analyses with 5000 resamples and 95% CI.

**Results:**

At one month postoperatively, 45.05% of patients experienced anxiety, 60.40% reported moderate-to-severe urinary incontinence, and 71.78% demonstrated low eHealth literacy. High adherence to PFMT was observed in only 36.63% of participants. UI severity was indirectly associated with anxiety through three pathways: eHealth literacy (*β* = 0.154, *P* < 0.01), PFMT adherence (*β* = −0.047, *P* < 0.05), and a sequential pathway involving both eHealth literacy and PFMT adherence (*β* = 0.038, *P* < 0.01). eHealth literacy accounted for 22.3% of the total effect, PFMT adherence for 6.8%, and their combined chain mediation effect for 5.5%.

**Conclusions:**

Urinary incontinence severity one month after prostate cancer surgery is a significant predictor of anxiety, exerting both direct and indirect effects. eHealth literacy and PFMT adherence function as important psychological and behavioral mediators, together explaining 34.6% of the total effect. Interventions aimed at enhancing eHealth literacy and promoting structured pelvic floor muscle training may help reduce postoperative anxiety and support recovery in this population.

## Introduction

Prostate cancer is the second most common cancer among men and ranks third in cancer-related mortality.^1^ For decades, radical prostatectomy has been considered the “gold standard” treatment for localized prostate cancer.^2^ However, postoperative complications—particularly incontinence after prostate treatment (IPT)—significantly impair patients'quality of life and have become a key focus in surgical innovation.^3^ With advances in early screening, standardized treatment, and comprehensive management, survival rates for prostate cancer have improved markedly. According to the American Cancer Society Prostate Cancer Survivorship Guidelines,^4^ the 10-year and 15-year relative survival rates are 97.8% and 91.4%. These trends in survival have been attributed to a combination of early detection, increasingly effective treatment of localized and advanced disease, lead-time bias (early diagnosis falsely appears to prolong survival), and overdiagnosis (often due to the widespread use of PSA screening). A longitudinal study on anxiety in patients with prostate cancer revealed that the prevalence of postoperative anxiety increased from 37.8% at baseline to 45.4% at 5 years,^5^ with higher anxiety levels consistently associated with poorer disease-free survival and overall survival. Elevated anxiety may influence tumor progression through neuroendocrine pathways, reduce treatment adherence, and is directly associated with poor prognosis.^6^ Furthermore, anxiety can lead to insomnia, appetite loss, reduced quality of life, increased risk of depression and suicide, thereby imposing additional burdens on families and society.^7^^,^^8^ From a treatment perspective, postoperative complications such as urinary incontinence are significant contributors to anxiety. At the psychosocial level, factors including male identity, optimism, and illness perception significantly influence anxiety levels.^9^ Variations in anxiety levels have been observed across different regions and durations since discharge. Developing intervention strategies based on a thorough understanding of anxiety mechanisms may help alleviate postoperative anxiety in these patients.^10^^,^^11^

In recent years, the impact of physical activity on anxiety and quality of life in patients with prostate cancer has become a research hotspot, yet high-quality evidence remains limited. A prospective cohort study of 33,908 adults over 11 years found that regular physical activity of any intensity could prevent depression, but had limited efficacy in preventing anxiety.^12^ Another study suggested that effective pelvic floor muscle training (PFMT) and exercise interventions can improve urinary incontinence and reduce anxiety, but did not explore the determinants or underlying mechanisms of anxiety changes.^13^ Overall, existing evidence confirms an association between physical activity and reduced anxiety; however, few studies have investigated the mechanisms through which exercise alleviates anxiety in patients with prostate cancer.

The severity of urinary incontinence affects PFMT adherence. A study on postoperative self-management behaviors in patients with prostate cancer found that patients' motivation improved when they observed the efficacy of PFMT.^14^ However, their adherence tended to decline once urinary incontinence symptoms alleviated. Additionally, maintaining long-term exercise motivation was challenging, particularly for individuals unaccustomed to regular physical activity. These findings suggest that PFMT adherence is associated with post-intervention improvements in urinary incontinence as well as patients' perceived severity of incontinence. Greater severity may serve as a stimulus for initiating training. While existing research has predominantly focused on the impact of PFMT adherence on incontinence severity, limited research has focused on the impact of urinary incontinence severity on patients' PFMT adherence and its mediating factors.

eHealth literacy refers to an individual's ability to access, understand, and evaluate health information through electronic media and to apply the acquired knowledge to address health-related issues.^15^ eHealth literacy, as an emerging concept in the information era, has become a research focus in recent years due to its association with patients' health behaviors and clinical outcomes. As a critical determinant of health behavior, eHealth literacy may exert either positive or negative effects, depending on factors such as regulatory efficacy, population characteristics, the nature of the health behavior itself, and the credibility of health information sources. Existing studies have demonstrated a positive correlation between eHealth literacy and health behaviors when supported by effective guidance from health care professionals.^16^ However, some studies have indicated that individuals with higher health literacy may exhibit greater skepticism toward health-related information, potentially leading to reduced vaccination willingness.^17^ Although research has shown no significant association between eHealth literacy and certain health behaviors (e.g., smoking, alcohol consumption),^18^ it is noteworthy that eHealth literacy serves as a critical determinant of PFMT quality, exerting a positive influence.^19^ Moreover, as PFMT is predominantly conducted at home with limited in-person guidance from health care professionals, eHealth literacy plays a particularly critical role in the rehabilitation process. Individuals with low health literacy are more prone to anxiety during disease recovery. Given that most PFMT-related health education relies on digital health information systems (e.g., mobile apps, online platforms), patients with higher educational attainment typically exhibit superior eHL proficiency. These individuals demonstrate enhanced capacity to access, interpret, and apply evidence-based health information, thereby improving exercise regimen comprehension and long-term adherence.^20^ eHL alone does not fully explain variations in PFMT adherence, with self-efficacy^21^ and social support systems^22^ being additional moderators. However, modifying objective factors such as social support remains challenging, whereas enhancing subjective factors (e.g., improving eHealth literacy) can deepen patients' disease-related knowledge and health belief adherence, thereby promoting self-efficacy and ultimately increasing PFMT adherence.

eHealth literacy has been shown to correlate with patient anxiety levels. During disease rehabilitation, individuals with low health literacy, particularly elderly patients, are prone to experiencing technology-related distress, which may exacerbate anxiety.^23^ For instance, patients with prostate cancer undergoing PFMT typically begin with video-guided instruction, followed by biofeedback therapy and mobile device monitoring. Upon treatment completion, they transition to home-based self-exercise regimens. However, the absence of on-site professional supervision often leads to technical difficulties, contributing to heightened anxiety.

eHealth literacy influences patient symptoms, while conversely, symptom severity may also reciprocally affect eHealth literacy. Importantly, eHealth literacy is not static, targeted interventions can enhance it, as demonstrated by improved levels among university students following structured training.^24^ Xue et al.^25^ conducted a latent class analysis of eHealth literacy among 558 stroke patients, identifying key predictors of its variability: educational attainment, health information sources, willingness to receive telemedicine, and comorbidity status. Notably, the presence of comorbidities emerged as a critical predictor, as patients with comorbid conditions actively sought health information, altering both their health-seeking behaviors and their perception of eHealth literacy. A systematic review highlighted that health-seeking behavior in cancer patients significantly shapes eHealth literacy,^26^ with evidence supporting its improvement through education. Longitudinally, symptom-driven proactive learning may elevate eHealth literacy. However, transient factors such as learning barriers or eHealth access challenges can lead patients to underestimate their eHealth literacy due to perceived knowledge or skill deficits.^27^ What is the mediating role of eHealth literacy in the relationship between somatic-psychological symptoms (e.g., urinary incontinence, anxiety) among patients with prostate cancer? Does the perceived severity of urinary incontinence modulate patients' self-reported eHealth literacy? These unresolved issues warrant rigorous exploration.

The Health Belief Model (HBM) was first proposed in the 1950s by American psychologists Hochbaum, Rosenstock, and Kegels to explain individuals' participation in tuberculosis screening programs, and was later expanded by Becker et al.^28^ As a psychological framework for understanding and predicting health-related behaviors, the HBM has since been widely applied in chronic disease management and health promotion interventions. The model posits that an individual's decision to engage in a health behavior is determined by their perception of threat associated with inaction and the perceived net benefit of taking action. Perceived threat is a composite of perceived susceptibility and perceived severity, while the net benefit is evaluated through a cost–benefit analysis of perceived benefits versus perceived barriers. However, in practical applications, the constructs of “perceived threat” and “benefit appraisal” within this model present significant measurement challenges. In recent years, with the rapid advancement of digital health technologies, researchers have extended the traditional HBM by incorporating eHealth literacy—a key cognitive capacity—as a central construct, leading to the development of an integrative theoretical framework known as the eHealth Literacy-based Health Belief Model (eHL-HBM).^29^ eHL-HBM extends beyond the traditional Health Belief Model (HBM), which primarily focuses on predicting behavior-outcome relationships, by incorporating eHealth literacy as a critical determinant. This model refines the construct of “perceived severity” and specifically identifies electronic health information as the principal target of “perceived barriers,” operationalizing this dimension through quantitative assessment. Within this theoretical framework, patients' perceptions of their health status and electronic health information directly influence their health behaviors, which in turn determine health outcomes. In the context of prostate cancer management, postoperative physiological symptoms such as urinary incontinence frequently induce clinically significant anxiety through a complex cognitive-behavioral-outcome interaction. During rehabilitation, patients encounter substantial eHealth information-related perceptions, wherein physical symptoms simultaneously modulate both the perception of eHealth literacy and health information acquisition, while eHealth literacy reciprocally affects psychological states. The eHL-HBM emphasizes a unidirectional “cognition→behavior→outcome” pathway, where cognition encompasses both “perceived severity” and “perceived barriers,” thereby providing a systematic theoretical foundation for elucidating the mediating mechanisms between symptom burden and psychological distress. When examining the anxiety-influencing pathways among urinary incontinence severity, eHealth literacy, and PFMT adherence, urinary incontinence severity corresponds to “perceived severity,” eHealth literacy aligns with “perceived barriers,” pelvic floor muscle training represents health behavior, and anxiety serves as the health outcome in this model.

**Fig. 1:**
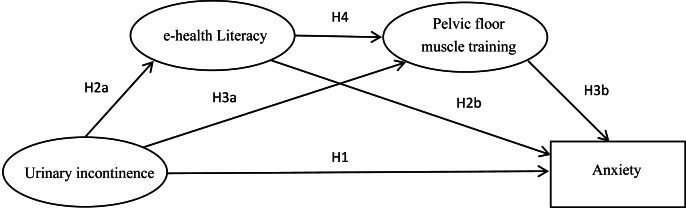
Hypothetical chain mediation model.

In summary, urinary incontinence severity and anxiety levels are high one month post-surgery, significantly impairing patients'quality of life.^30^ eHealth literacy and PFMT adherence constitute critical determinants influencing postoperative physical and psychological outcomes in patients with prostate cancer. Previous studies have predominantly examined bivariate relationships between isolated variables and either anxiety or urinary incontinence severity,^31^ failing to investigate mediating roles of variables between physical and psychological symptoms or elucidate the interrelationships among symptoms, informational competence, health behaviors, and emotional states. Notably, the potential mechanistic role of eHealth literacy in mediating the body-mind symptom interaction remains unexplored. Urinary incontinence and anxiety represent the most prevalent postoperative physical and psychological symptoms, respectively, whose bidirectional influence complicates targeted intervention development. This study aims to systematically delineate the psychobehavioral mechanisms through which urinary incontinence affects anxiety in patients with prostate cancer, clarify the psychological transformation process of symptom distress, and establish both theoretical foundations and modifiable targets for precision psychological interventions. Therefore, this study adopted the eHL-HBM framework to investigate the interplay between urinary incontinence severity, eHealth literacy, PFMT adherence, and anxiety, aiming to provide a comprehensive understanding of the psychological mechanisms following prostate cancer surgery. This study proposes the following hypotheses: eHealth literacy mediates the relationship between incontinence severity and anxiety; PFMT adherence mediates the relationship between incontinence severity and anxiety; and eHealth literacy and PFMT adherence jointly form a serial mediation model in the pathway from incontinence severity to anxiety. By constructing a serial mediation model, This study aims to elucidate the underlying mechanisms of anxiety in patients with prostate cancer, providing theoretical support and decision-making guidance for the development of clinical interventions. The hypothetical model used in this study is shown in Fig. 1.

H1 Urinary incontinence has a direct effect on postoperative anxiety in patients with prostate cancer.

H2 eHealth literacy mediates the relationship between postoperative urinary incontinence and anxiety in patients with prostate cancer.

H3 PFMT adherence mediates the association between postoperative urinary incontinence and anxiety in patients with prostate cancer.

H4 eHealth literacy and PFMT adherence jointly mediate the relationship between postoperative urinary incontinence and anxiety through a serial mediation pathway.

## Methods

### Study design and participants

This study employed a convenience sampling method to recruit patients with prostate cancer hospitalized at a tertiary hospital in Wuhan, China, between December 2023 and December 2024. Inclusion criteria were: (1) pathologically confirmed localized prostate cancer;^32^ (2) age ≥ 18 years; (3) discharged after undergoing radical prostatectomy and returning to the hospital for follow-up one month postoperatively; and (4) capable of normal communication and providing informed consent to participate voluntarily. Exclusion criteria included: (1) visual or hearing impairments that would interfere with study procedures; (2) presence of other malignant tumors; or (3) a history of psychiatric disorders or cognitive impairment. According to prior research,^33^ the recommended sample size for structural equation modeling is between 200 and 600, which provides adequate statistical power and model stability. A priori power analysis for serial mediation was conducted using Monte Carlo simulation^34^ with predicted effect sizes based on previous studies on cancer survivorship and self-management (e.g., eHealth literacy → adherence: *β* ≈ 0.35). Results indicated that a sample size of 180 would provide 80% power to detect a small-to-medium serial mediation effect (*f*^2^ = 0.15) at *α* = 0.05. To account for potential missing data, we aimed to recruit at least 200 participants. In fact, a total of 240 participants were enrolled in this study, and 202 valid responses were ultimately obtained. A total of 38 questionnaires were excluded due to incomplete responses (*n* = 11) or excessive missing data (*n* = 27).

### General information questionnaire

A general data questionnaire is used to collect the basic information of patients, using self-prepared questionnaire, including age, education, education, hospitalization days, etc.

### PFMT adherence scale

PFMT adherence was assessed using the PFMT Adherence Scale developed by Chen et al.^35^ The Chinese version of this questionnaire has demonstrated good internal consistency and test-retest reliability, with a Cronbach's *α* coefficient of 0.81. The scale comprises three dimensions: exercise duration, exercise frequency, and concordance between performed PFMT and the instructed technique. The first two items are rated on a 5-point Likert scale, while the third item is evaluated using a visual analog scale (VAS) ranging from 0 (“no adherence at all”) to 10 (“complete adherence”). The total score is the sum of the three items, yielding a possible range of 2–21. Adherence levels were categorized as follows: poor adherence (total score < 7), moderate adherence (score 7–14), and high adherence (score 15–21). In this study, the average item score was 10.82 (standard deviation = 5.67), with pairwise inter-item correlations spanning from 0.79 to 0.94, reflecting substantial item consistency.

### Electronic health literacy scale (eHealth literacy scale, eHEALS)

The Chinese version of the eHealth Literacy Scale^36^ was used. This scale consists of 8 items across three dimensions: ability to apply online health information and services (5 items), appraisal ability (2 items), and decision-making ability (1 item). A 5-point Likert scale was employed, ranging from “strongly disagree” to “strongly agree,” with responses scored from 1 to 5. The total score ranges from 8 to 40, with higher scores indicating higher levels of eHealth literacy. The eHEALS uses a 5-point Likert scale with three classification levels: low (≤ 26), moderate (27–32), and high (≥ 33)." The scale demonstrated a Cronbach's α coefficient of 0.913 and a split-half reliability of 0.946, indicating excellent internal consistency and reliability. In this study, the Cronbach's α for the eHealth literacy scale was 0.710, which is within the acceptable range.

### International advisory committee on the Incontinence Questionnaire-Short Form (ICIQ-SF)

The scale was developed by the International Continence Society (ICS) in 2004. The Chinese version has been validated in patients following radical prostatectomy, demonstrating good reliability and validity.^37^ The questionnaire comprises four items: frequency of urine leakage (never = 0; approximately once per week or less = 1; 2–3 times per week = 2; approximately once daily = 3; several times daily = 4; continuous leakage = 5), amount of urine leakage (no leakage = 0; small amount = 2; moderate amount = 4; large amount = 6), impact of leakage on daily life (rated on a 0–10 numerical rating scale, where 0 = “no impact” and 10 = “extreme impact”), and duration of leakage. The total score ranges from 0 to 21, with higher scores indicating greater severity of urinary incontinence. Incontinence severity is determined by summing the scores for frequency, amount, and impact on life: mild incontinence (0–7 points), moderate incontinence (8–14 points), and severe incontinence (15–21 points). The scale has a Cronbach's α coefficient of 0.81, and the Chinese version shows high agreement with urodynamic diagnosis (Kappa = 0.77), indicating strong internal consistency and criterion-related validity.^38^ In the present study, the ICIQ-SF had good reliability, with Cronbach's α exceeding 0.846.

### Generalized anxiety scale (Generalized Anxiety Disorder 7, GAD-7)

The scale was developed based on the Diagnostic and Statistical Manual of Mental Disorders, Fourth Edition (DSM-IV).^39^ In Chinese hospitalized patients, it demonstrates good internal consistency with a Cronbach's α coefficient of 0.898 and excellent test-retest reliability of 0.856.^40^ The scale consists of 7 items, each rated on a 4-point scale (0 = “not at all”; 1 = “on several days”; 2 = “on more than half of the days”; 3 = “on nearly every day”), yielding a total score ranging from 0 to 21. Severity levels are categorized as follows, 0–4 points: No clinically significant anxiety; 5–9 points: Mild anxiety; 10–14 points: Moderate anxiety; ≥ 15 points: Severe anxiety.^41^ In the present study, the GAD-7 had good reliability, with Cronbach's α exceeding 0.871.

### Data collection

We established a research team consisting of one medical doctor and four master's degree candidates in nursing. All team members completed systematic training to ensure thorough understanding of the research protocols and strict adherence to necessary precautionary measures during questionnaire administration, including the use of standardized verbal instructions and verification of data completeness. During the hospitalization period, specialized nurses in pelvic floor rehabilitation delivered health education to patients through printed materials and individual verbal instruction, and explicitly informed patients of the specific follow-up arrangements one month after discharge. On the day of discharge, the attending physicians documented the outpatient follow-up schedule, location, and relevant precautions in the discharge summaries to ensure patients were fully informed of the follow-up requirements. At the one-month postoperative follow-up visit, the research team administered the questionnaires upon obtaining written informed consent from the patients. The average time required to complete the questionnaire was limited to 20–30 minutes. As a token of appreciation, commemorative gifts were provided to participating patients and their families. A total of 240 patients with prostate cancer were surveyed, resulting in 202 valid responses, with an effective response rate of 84.17%.

### Statistical analysis

Questionnaires were assigned unique identification numbers and double-entered into Excel 2019 by two independent data entry clerks, with cross-checking performed to ensure accuracy. Descriptive statistics were used to summarize the study variables: categorical variables were presented as frequencies and percentages, while continuous variables were described using means and standard deviations. Data were analyzed with SPSS 25.0. Pearson correlation analysis was conducted to examine the relationships among variables. Structural equation modeling (SEM) was further performed using Amos 24.0 to test the hypothesized model. The bootstrap method with 5000 resamples was applied to assess model fit and estimate bias-corrected 95% confidence intervals for direct, indirect, and total effects. The statistical significance level was set at *α* = 0.05.

## Results

### Data processing

The data utilized in this study consist entirely of continuous variables. However, variations exist in the dimensions and scoring criteria of the measurement scales. During the data analysis phase, the original data were input directly; however, Z-score transformation, a built-in function in AMOS, was applied to standardize the data. In the presentation of results, the standardized output was selected, with particular emphasis placed on interpreting the Standardized Estimates section.

### Validity and reliability/rigour

Following data collection, all variables were screened for missing data and univariate outliers to ensure data quality prior to analysis. All variables met the assumption of normality. The Harman single-factor test^42^ was used to assess the common method bias. The results showed that ten factors had eigenvalues > 1, with the first factor explaining 36.45% of the variance, which is less than the critical standard of 40%. After data collection, we performed checks for missing data and outliers. Since no missing values were detected during systematic screening (using [e.g., Python's pandas.isnull(.)/SPSS Missing Value Analysis]), no data exclusion or imputation procedures were required, and all variables followed a normal distribution. Besides, the main variables in this study were assessed with previously validated scales and each scale had a Chinese version and showed an acceptable internal consistency.

### Demographics and clinical characteristics

A total of 202 patients diagnosed with prostate cancer were included in the study, with ages ranging from 48 to 82 years (mean age = 66.89, SD = 6.95 years). The duration of hospitalization varied from 4 to 22 days (mean = 8.20, SD = 2.83 days), and the operative time ranged from 57 to 345 minutes (mean = 158.97, SD = 42.58 minutes). Comprehensive demographic and clinical data of the patients are presented in Table 1.

**Table 1:** General demographic and disease-related information.

Variable	*n*	%
**Age (years)**		
< 60	26	12.87
From 60 to 69	106	52.48
70-79	66	32.67
Admedia 80	4	1.98
**Education level**		
Primary and below	21	10.39
Junior high school	83	41.09
Senior high school	49	24.26
University and above	49	24.26
**Lifestyle**		
Living with family	159	78.71
Living alone or otherwise	43	21.29
**Current address**		
Municipalities	146	72.28
Township or rural	54	27.72
**Surgical approach**		
Robot-assisted laparoscopic surgery	180	89.11
Laparoscopic surgery	22	10.89

### The score of each variable

PFMT adherence, e-health literacy level, urinary incontinence score and anxiety score of patients with prostate cancer are shown in Table 2.

**Table 2:** Descriptive Statistics of study variables (*N* = 202).

Variable	Severity score	*n* (%)	Total score (Mean ± SD)
Urinary Incontinence severity	Mild	80 (39.60)	9.69 ± 4.74
	Moderate	88 (43.56)	
	Severe	34 (16.83)	
eHealth literacy	Low	145 (71.78)	5.03 ± 4.80
	Moderate	54 (26.73)	
	High	3 (1.49)	
Pelvic floor muscle training adherence	Low	53 (26.24)	10.82 ± 5.67
	Moderate	75 (37.13)	
	High	74 (36.63)	
Anxiety level	No anxiety	111 (54.95)	20.92 ± 8.32
	Mild	48 (23.76)	
	Moderate	34 (16.83)	
	Severe	9 (4.46)	

SD, standard deviation.

### Relationships among variables

Correlation of PFMT adherence, e-health literacy, urinary incontinence score, and anxiety score in patients with prostate cancer, Refer to Table 3 for further details. At one month postoperatively, anxiety levels were significantly positively correlated with urinary incontinence severity (*r* = 0.724, *P* < 0.01), and negatively associated with eHealth literacy (*r* = −0.561, *P* < 0.01) and PFMT adherence (*r* = −0.229, *P* < 0.01).

**Table 3:** Intercorrelations among key variables (*N* = 202).

Variable	Urinary incontinence	Anxiety	PFMT	eHealth Literacy
Urinary incontinence	1			
Anxiety	0.724^b^	1		
PFMT adherence	0.144^a^	−0.229^b^	1	
eHealth literacy	−0.485^b^	−0.561^b^	0.308^b^	1

PFMT: pelvic floor muscle training;

a *P* < 0.05.

b *P* < 0.01.

### Mediator model

**Fig. 2:**
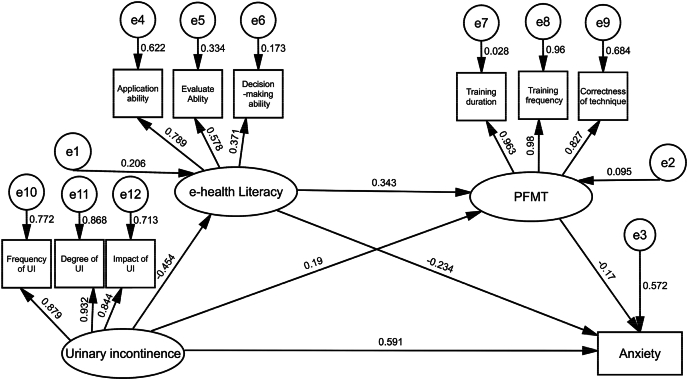
Model diagram of the chain mediation effect.

Model fit indices indicated good overall fit: *χ*^2^/df = 1.347, RMSEA = 0.042 (90% CI [0.021, 0.061]), CFI = 0.984, TLI = 0.971, and SRMR = 0.048. These values meet or exceed conventional criteria for acceptable model fit (CFI, TLI > 0.95; RMSEA, SRMR < 0.08).^43^ No post-hoc modifications were made to the model based on modification indices, as the a priori theoretical model demonstrated adequate fit. The final mediation model is presented in Fig. 2.

### Assessment of model stability

Although the dataset was complete and no severe distributional issues were detected, the model may still be sensitive to sampling variability or extreme cases. To evaluate stability, we conducted two checks: (1) The consistency of significant indirect effects across 5000 bootstrap samples was examined to assess replicability; (2) Sensitivity to outliers was evaluated by identifying cases with standardized residuals exceeding ± 3.29, removing them, and re-estimating the model to compare changes in key path coefficients. Stability was supported if the direction and significance of paths remained unchanged.

### Analysis of pathways and examination of mediation effects

Bootstrap Method test (setting repeated random sampling 5000 times) was used to calculate 95% CI. The results showed that the 95% confidence intervals of direct and indirect effects of electronic health literacy and PFMT adherence on patient anxiety did not include zero, indicating that some mediation effect was established and accounted for 34.6% of the total effect. The results of mediation effect test are shown in Table 4.

**Table 4:** Bootstrap analysis of mediation effect significance.

Effect path	Effect value	Standard error (SE)	Bootstrap CI (95%)	*P*	Effect size (%)
Urinary incontinence → eHL → anxiety	0.154	0.042	[0.080,0.240]	0.004^b^	22.3
Urinary incontinence → PFMT adherence → anxiety	−0.047	0.018	[-0.085,-0.006]	0.028^a^	6.80
Urinary incontinence → eHL → PFMTadherence → anxiety	0.038	0.015	[0.012,0.070]	0.001^b^	5.50
Total indirect effect	0.145	0.040	[0.075,0.225]	0.001^b^	34.6
Direct effect	0.591	0.121	[0.435,0.697]	0.001^b^	65.4
Total effect	0.736	–	[0.600,0.870]	< 0.001^c^	100

eHL, eHealth literacy; PFMT, pelvic floor muscle training; CI, confidence interval; Bootstrap, 5000 resamples. Significant if 95% CI excludes zero.

a *P* < 0.05.

b *P* < 0.01.

c *P* < 0.001.

### Sensitivity and stability assessment

Given the moderate sample size, we acknowledged the potential for model instability, particularly in estimating complex mediation paths. To assess the robustness of our findings, we employed bias-corrected bootstrap confidence intervals (5000 resamples) for all path coefficients and indirect effects. Additionally, we compared the fit of the hypothesized model with an alternative model in which the order of mediators was reversed, to evaluate directional specificity. These sensitivity checks support the stability and theoretical consistency of the reported results.

## Discussion

This study confirmed the initial hypotheses, demonstrating significant associations among urinary incontinence severity, eHealth literacy, PFMT adherence, and anxiety in patients with prostate cancer. Mediation analyses revealed a multilevel mechanism: both eHealth literacy and PFMT adherence independently mediated the incontinence and anxiety link, with an additional serial pathway (urinary incontinence → eHealth literacy → PFMT adherence → anxiety). This finding provides empirical support for the eHL-HBM model and deepens the understanding of post-prostatectomy psychological response mechanisms. It reveals that patients' physical health information influences self-perceived cognitive abilities (e.g., information acquisition and comprehension), which in turn promotes health behaviors (e.g., rehabilitation training), ultimately affecting mental health outcomes. This forms a dynamic chain of “physical function → cognition → behavior → psychological state”. For prostate cancer survivorship, this serial mediation signifies a shift from isolated symptom control to an integrated model linking digital competence, health behaviors, and psychophysiological outcomes—laying the groundwork for patient-centered, precision rehabilitation. More broadly, our results extend the biopsychosocial framework within Chronic Disease Self-Management, revealing a significant mismatch between eHealth needs and information literacy among patients with prostate cancer, while also highlighting the synergistic role of digital literacy and behavioral engagement and offering a multidimensional perspective on anxiety etiology in long-term survivors.

The study found that postoperative urinary incontinence in patients with prostate cancer has a direct predictive effect on anxiety. Recovery from post-prostatectomy incontinence varies according to surgical technique, age, and other clinical factors, with most patients experiencing gradual improvement within 3–12 months. The early postoperative period—particularly around one month after catheter removal—is typically characterized by the most severe incontinence.^44^ Therefore, our study focused on patients at one month post-surgery, and the observed severity of incontinence aligns with findings from Qiao et al.^45^ In this cohort, the prevalence of anxiety exceeded the previously reported rate of 45.4%,^46^ which may be attributed to both the demographic characteristics of our sample and the timing of assessment. Elevated anxiety is common after prostate cancer treatment and may persist for up to 1 or even 5 years, driven by factors such as cancer-related fatigue, urinary incontinence, and sexual dysfunction.^47^ Urinary incontinence can lead to feelings of shame, social withdrawal, and threats to masculine identity, exacerbating psychological distress and contributing to the development of anxiety.^48^ Clinicians have long sought to alleviate anxiety by improving urinary continence. Advances in surgical techniques,^49^ early multimodal physical rehabilitation,^50^ and the use of artificial urinary sphincters^51^ offer promising strategies to mitigate both incontinence and its associated psychological burden.

eHealth literacy was found to mediate the relationship between urinary incontinence severity and anxiety in patients with prostate cancer after surgery. As shown in Table 2, the mean eHealth literacy score was 5.03 ± 4.80, patients with low eHealth literacy accounted for 71.78% of the sample, which is consistent with previous findings in similar populations.^52^ Patient age ranged from 48 to 82 years (mean: 66.89 ± 6.95), and the largest educational group had junior high school or vocational secondary education (*n* = 83, 41.09%) (Table 1). The e-health literacy level of patients with prostate cancer is influenced by factors such as age and education.^53^ Advanced age and lower educational attainment are predictors of low health literacy. Under normal circumstances, patients may hardly notice this issue. However, when urinary incontinence becomes severe, patients actively seeking e-health information may become aware of their inadequate e-health literacy, which in turn exacerbates their anxiety. Our findings suggest that enhancing eHealth literacy may strengthen patients' understanding of disease management and improve decision-making capacity, thereby mitigating anxiety. As early as 2010, the U.S. Office of the National Coordinator for Health Information Technology released the National Action Plan to Improve Health Literacy, advocating for health systems to be designed with health literacy in mind and promoting patient engagement in self-management through digital tools.^54^ Similarly, China's 14th Five-Year Plan for National Health Informatization (2022) explicitly emphasizes improving digital health literacy and expanding access to digital health services.^55^ However, translating policy into practice remains a significant challenge. To improve eHealth literacy among prostate cancer survivors, several strategies are recommended: (1) integrating the Health Belief Model into clinical education programs to enhance perceptions of behavioral benefits and self-efficacy;^56^ (2) implementing theory-guided interventions such as cognitive-behavioral therapy and decision aids to reduce anxiety, particularly among older and less-educated patients;^57^^,^^58^ (3) Addressing the “digital divide” by simplifying information delivery—using visual guides, instructional videos, and plain language—to prevent information overload and associated psychological distress.^59^

PFMT adherence mediated the relationship between urinary incontinence severity and anxiety in patients with prostate cancer following surgery. As shown in Table 3, PFMT adherence is positively correlated with urinary incontinence but negatively correlated with anxiety levels. Table 4 further indicates that PFMT adherence exerts a significant partial mediating effect, accounting for 6.8% of the total association. These findings suggest that PFMT not only improves pelvic floor muscle strength but also exerts beneficial psychological effects. From a neurobiological perspective, physical activity may promote lactylation of synaptic proteins in the cerebral cortex, enhancing stress resilience and reducing anxiety.^60^ Clinically, exercise has been shown to slow biochemical progression in prostate cancer and alleviate postoperative psychological distress, including anxiety.^61^ However, adherence to PFMT remains suboptimal among prostate cancer survivors. This is likely attributable to factors such as exercise-related discomfort, lack of structured guidance, and unsupportive rehabilitation environments.^62^ To enhance PFMT adherence and thereby reduce anxiety, the following strategies are recommended: (1) implementing long-term interventions with continuous monitoring, such as remote tracking and regular feedback systems;^63^ (2) establishing supportive communication platforms to foster family involvement and peer education;^64^ (3) standardizing training protocols and outcome assessments, while simplifying exercise procedures to reduce implementation burden.^65^

A serial mediation effect was observed in which eHealth literacy and PFMT adherence sequentially link urinary incontinence severity to anxiety levels, as illustrated in Fig. 2. As shown in Table 4, the mediating effect of eHealth literacy was significantly greater than that of PFMT adherence (*P* < 0.01). This finding aligns with prior research demonstrating that eHealth literacy and social support jointly mediate the relationship between depression and quality of life in older adults.^66^ Theoretically, this serial pathway supports the eHealth Literacy-based Health Belief Model, an extension of the traditional Health Belief Model^67^ into the digital health era.^68^ The eHL-HBM posits that engagement in health-promoting behaviors depends not only on perceived susceptibility, severity, and benefits, but also critically on an individual's capacity to access, understand, evaluate, and apply electronic health information. Individuals with higher e-health literacy may exhibit lower health compliance when encountering uncertain electronic health information.^17^ However, for patients with prostate cancer with low e-health literacy, the fact that pelvic floor muscle training can alleviate urinary incontinence is a well-established health claim. According to the eHL-HBM model, severe urinary incontinence prompts patients to actively seek electronic health information. Upon recognizing their limited ability to evaluate such information, they are motivated to improve their e-health literacy—enhancing their capacity to acquire, comprehend, and apply health-related knowledge. This, in turn, encourages consistent pelvic floor muscle training, ultimately reducing anxiety. Moreover, postoperative anxiety related to incontinence may also stem from suboptimal preoperative communication, limited shared decision-making, and subsequent decisional regret.^69^ Therefore, effective mitigation of anxiety requires comprehensive, individualized support from both societal and health care systems.^70^ At the societal level, age-friendly digital health platforms and intelligent training assistive devices should be developed, integrating lifestyle interventions and behavioral therapies to support self-management.^71^ At the clinical level, a graded management system for treatment-related adverse effects should be established to enable early identification of urinary incontinence and anxiety risks.^72^ Psychological screening should be routinely incorporated into follow-up care to facilitate timely intervention and management.^73^ Furthermore, advancing intelligent surgical platforms—integrating robotic surgery, intraoperative navigation, and functional monitoring—may reduce the incidence of incontinence through precision anatomical preservation.^74^

While this study yields significant findings that inform future research, several limitations warrant consideration. Despite strict ethical compliance and informed consent, survey procedures may inadvertently induce patient anxiety, potentially compromising data validity. The absence of stratified analyses precludes examination of confounding factors (e.g., health literacy, psychological comorbidity, or illness perception) on PFMT adherence and anxiety. Furthermore, the cross-sectional design cannot establish temporal precedence or dynamic interactions between mediators and outcomes. Future studies should incorporate stratified analyses with repeated measurements at multiple time points (e.g., baseline, 3-, 6-, and 12-month follow-ups), utilize analytical approaches such as cross-lagged panel models or growth curve models to examine temporal relationships, and implement more objective assessment tools for both pelvic floor muscle training adherence and eHealth literacy levels. Additionally, integrating qualitative research methodologies would provide deeper insights into patient experiences. This multidimensional approach would enable comprehensive investigation of the dynamic interactions among urinary incontinence, eHealth literacy, pelvic floor muscle training adherence, and anxiety, ultimately facilitating the development of more targeted personalized intervention strategies.

### Implications for nursing practice and research

This study has important theoretical and practical implications. Grounded in the eHealth Literacy-based Health Belief Model, it supports a shift from a symptom-centered care approach to a more behavior-centered and holistic model of patient care, thereby empowering patients to become active participants in their recovery process. In clinical practice, nurses should routinely assess patients' eHealth literacy and provide tailored support to help them effectively access, understand, and apply digital health information. Additionally, structured nursing interventions should be designed to promote sustained and correct performance of pelvic floor muscle training, incorporating goal setting, motivational enhancement, and regular follow-up. In nursing education, digital health competencies should be integrated into the curriculum to prepare future nurses to deliver technology-enabled, patient-centered care. From a research perspective, future studies should employ longitudinal designs and multi-center, large-sample investigations to further validate the identified mediating pathways. More comprehensive theoretical models could incorporate psychosocial variables such as self-efficacy, illness acceptance, social support, and quality of patient-nurse communication. Importantly, there is an urgent need for nurse-led interventional studies to evaluate the effectiveness of integrated interventions that simultaneously improve eHealth literacy, enhance PFMT adherence, and reduce both urinary incontinence and anxiety. Such efforts will strengthen the role of the nursing profession in building efficient, technology-empowered care systems for cancer survivors.

### Limitations and future directions

The limitations of this study should be acknowledged. First, all participants were recruited from a single tertiary hospital in China, which may introduce selection bias and limit the generalizability of the findings to other populations, particularly those in different health care systems or cultural contexts. Future multicenter studies are warranted to validate the conclusions of this research. Second, although we controlled for several sociodemographic and clinical variables, potential influencing factors such as cultural beliefs, health communication styles, and personality traits (e.g., neuroticism or self-efficacy) were not fully accounted for in the analysis. In particular, the study population consisted exclusively of patients with prostate cancer, who were predominantly elderly. This distinct demographic characteristic may limit the generalizability and applicability of our proposed mediation model to broader populations. Third, the cross-sectional design precludes causal inference; therefore, the observed associations between urinary incontinence severity, eHealth literacy, PFMT adherence, and anxiety levels reflect only concurrent relationships at the time of assessment. Longitudinal or interventional studies are needed to examine temporal and causal dynamics. Fourth, the measures used—particularly the eHealth literacy scale—were developed in Western contexts and may not fully capture culturally specific aspects of digital health engagement in Chinese populations. This raises questions about the cross-cultural validity and applicability of the constructs. Finally, while our model demonstrated acceptable fit, future studies could employ Monte Carlo simulations to evaluate parameter recovery and statistical power for this type of serial mediation model in samples of similar size.

## Conclusions

This study determined the relationship between urinary incontinence severity, e-health literacy, PFMT adherence, and anxiety levels. The anxiety of patients with prostate cancer was at a moderate level one month after surgery, the incidence of anxiety was high, and the severity of urinary incontinence affected the anxiety level of patients. The severity of urinary incontinence was found to have a positive correlation with patients' anxiety levels, while simultaneously exerting a negative influence on anxiety through the mediating factors of e-health literacy and PFMT adherence. This indicates a chain mediation effect, with the mediating role of e-health literacy being more pronounced than that of PFMT adherence. Furthermore, patients' anxiety levels can be mitigated directly by reducing the severity of urinary incontinence, as well as indirectly through enhancements in e-health literacy and adherence to pelvic floor muscle training.

## CRediT authorship contribution statement

QXQ: conceived and designed the study and wrote the initial draft of the manuscript. JL: conceived and designed the study and revised the first draft of the paper. DZ: contributed to manuscript text optimization and picture modification. YMZ: collected and verified data. XQX: Collect and verify data. FY: Contribute to data retrieval and collation. LHH: Supervision, Methodology, Review & editing. XQC: contributed to the study design, provided critical revisions for important intellectual content, and gave final approval of the version to be published. All authors have read and approved the final manuscript.

## Ethics statement

This study was approved by the Ethics Committee of Tongji Hospital (Approval No. TJ-IRB20231165) and was conducted in accordance with the 1964 Helsinki Declaration and its later amendments or comparable ethical standards. All participants provided written informed consent.

## Data availability statement

The data that support the findings of this study are available from the corresponding author, XQC, upon reasonable request. The data are not publicly available due to their containing information that could compromise the privacy of our participants.

## Declaration of generative AI and AI-assisted technologies in the writing process

No AI tools/services were used during the preparation of this work.

## Funding

This study was supported by Tongji Hospital, Tongji Medical College, Huazhong University of Science and Technology (Grant No. 2024D38). The funders had no role in considering the study design or in the collection, analysis, interpretation of data, writing of the report, or decision to submit the article for publication.

## Declaration of competing interest

The authors declare no conflicts of interest.
